# Antinociceptive effects of vitamin B-complex: A behavioral and histochemical study in rats

**DOI:** 10.1016/j.ibneur.2023.09.005

**Published:** 2023-10-10

**Authors:** Shahab A. Zarei, Mina Shahriari-Khalaji, Ian Max Andolina, Gila Behzadi

**Affiliations:** a Center for Excellence in Brain Science and Intelligence Technology (Institute of Neuroscience), Chinese Academy of Sciences, 320 Yue Yang Road, Shanghai, China; b Department of Physiology, School of Medicine, Shahid Beheshti University of Medical Sciences, Tehran, Iran; c State Key Laboratory for Modification of Chemical Fibers and Polymer Materials, College of Materials Science and Engineering, Donghua University, Shanghai 201620, China

**Keywords:** B-vitamins, Neurobion, Antinociception, Inflammatory pain, Formalin test, *c-Fos*, NADPH-d

## Abstract

B-vitamins have been evaluated as a useful adjuvant therapy to treat pain. In spite of clinical and experimental evidence indicating the analgesic effect of B-vitamins, few studies have investigated their effect on aspects of the inflammatory pain response. In the present study, we investigated the analgesic effect of chronic application of B-complex vitamins (Neurobion) using an inflammatory experimental pain model in rats. Nociceptive behavioral responses were evaluated in male Wistar rats after plantar injection of formalin, comparing the treatment group (TG) with Neurobion pretreatment to the control group (CG) without the pretreatment. In addition, neuronal activity in the central pain pathway was evaluated using *c-Fos* immunohistochemical reactivity and NADPH-d histochemistry. A highly significant reduction of painful behaviors such as licking and flinching were observed in TG, especially during the secondary phase of the formalin test compared to CG. Results suggest that long-term pre-treatment using Neurobion can have a beneficial effect in reducing the chronic phase of pain. In addition, we observed a downregulation of *c-Fos* and NADPH-d in dorsal spinal neurons, suggesting that the antinociceptive effect induced by Neurobion could be due to a suppression of nociceptive transmission at the spinal level, particularly in the afferent regions of the dorsal spinal horn, which these neurons utilizing nitric oxide at least as one of their pain neurotransmitters.

## Highlights

• Neurobion pretreatment reduces pain-related behavior in a formalin test.
• Neurobion downregulates c-Fos and NADPH-d reactivity in dorsal spinal neurons.
• Neurobion’s analgesic effect suppresses pain transmission at the spinal level.
• Active dorsal horn neurons use NO at least as one of their pain neurotransmitters.
• Reducing neuronal activity at the site of afferent fibers links to analgesia.

## Graphical Abstract

**Figure:**
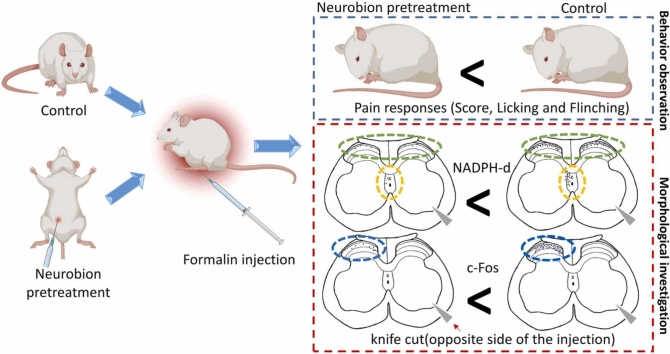

## 1 Introduction

B vitamins should be consumed daily as part of our dietary intake due to their critical and wide-ranging utility in the body, particularly considering that they are not stored by our body. The deficiency of these vitamins can cause pathophysiological conditions including epilepsy, carpal tunnel syndrome, chronic pain, sleep disorders, and autoimmune disorders (Gominak, 2016). In addition to treating deficiencies in the body, B-vitamins appear to engender analgesic effects in doses higher than the daily requirement. This suggests their usefulness in the treatment of pathophysiological pain disorders. Since impressive clinical results (REDMOND, 1957, Steinberg, 2007) were first observed in the 1950s, researchers have maintained a focus on the pain-relieving effects of B vitamins. The anti-inflammatory effects of B-vitamins have also been broadly studied in animal models, including mechanical allodynia and neuropathic pain (Menezes et al., 2017).

As well, it has been assumed that the synergistic effect of vitamin B1, B6, and B12 (as B-complex) could be more effective in reducing pain, with no risk of side-effects even at the high doses of consumption. This is the reason why it has been extensively used in clinical pain conditions. Although multiple mechanisms have been suggested to explain the analgesic and anti-inflammatory effects of B-complex vitamins (Hurt et al., 2012, Song et al., 2009, Thériault et al., 2014, Erfanparast et al., 2017, Hosseinzadeh et al., 2012), few investigations have been reported on the effects of B-complex vitamins on analgesia. Fewer studies have investigated their effects on the different aspects of the inflammatory pain response, and the exact pain reduction mechanism caused by B vitamins remains unknown.

The formalin test in rodents is extensively used to induce and model acute and chronic inflammatory pain (Bannon and Malmberg, 2007, Dzoyem et al., 2017). It has been reported that formalin injection is a valid and reliable model that is highly sensitive in the testing of varying classes of analgesic drugs (Murray et al., 1988, Berge, 2011, Hunskaar et al., 1985, Liu et al., 2019).

The pain signaling process is a complicated one involving a large number of neurotransmitters, molecules, and chemicals in the body. Nitric oxide (NO), a free radical diffusible gas molecule that is synthesized on demand (in response to inflammatory stimulation) with a half-life of few seconds, is one of the most important neurotransmitters involved in neuropathic pain in the spinal cord. It also plays an important role in the guidance and modulation of signals related to pain, neuronal protection, neurotoxicity, neuroplasticity and persistent inflammation (Abramson, 2008). Nitric oxide synthase (NOS) produces NO by oxidation of L-arginine, the NO precursor, to L-citrulline. The essential reductive cofactor NADPH or the NOS enzyme involved in NO synthesis can be used as a marker of NO presence (Janicki and Jeske-Janicka, 1998). Many researchers have reported that inhibition or knockout of NOS results in pain persistence (Schmidtko, 2015).

The expression of the proto-oncogene protein *c-Fos*, encoded by a member of the immediate-early gene family, is an important neurophysiological response to a variety of painful stimuli. *c-Fos* is rapidly induced to control the expression of other genes whose products are involved in long-term changes in neuronal excitability, and can be detected using immunohistochemistry (Li et al., 2019). During nociceptive transmission, Fos-like immunoreactivity is reported through the spinal dorsal horn up to the cerebral cortex, indicating the neural cascade of signal transduction. Therefore, mapping *c-Fos* expression in the areas is a valid and powerful tool to trace populations of nociceptive neurons responding to pain (Chen et al., 2013, Hossaini et al., 2014).

Given the promising results reported in clinical trials on the anti-inflammatory and pain-reducing effects of B-complex vitamin supplements, in this study we utilized the formalin test in rats to evaluate a pain model that is closest to clinical pain. We aimed to determine whether the Neurobion B-complex modified expressed pain behavior, and if so, delineate in which phase (acute and/or chronic) any effect was seen. Furthermore, we chose to delineate any such effects on both NO-producing neurons and *c-Fos* expression in the dorsal horn of the lumbar spinal cord and supra-spinal centers that are involved in pain processing.

## 2 Materials and methods

### 2.1 Animals and treatment

Male Wistar rats with an average weight of 195 ± 15 g were the model animal used in this study. All animals were kept single-housed, with free access to food (standard chow diets, without any dietary deficiency in B vitamins) and water, and maintained at a constant temperature of 22 ± 2 °C in a laboratory with a 12 h/12 h light cycle. The animal research fully complied with the principles and protocols of working with animals and experiments, guidelines for animal care established by the International Association for the Study of Pain (IASP), and consistent with NIH and our institute’s Animal Ethics Committee guidelines for studies in animals.

The rats were divided into three groups and each group contained eight rats (n = 8); the number of rats was chosen based on previously published studies (Baulmann et al., 1999, Hwang et al., 2008, Luongo et al., 2013, Ionov et al., 2019). For behavioral observation and histochemical evaluation, six and four rats were chosen respectively, and randomly from each group. The first experimental group received injections of 50 µl of formalin subcutaneously on the plantar surface of the right hind paw using needle number 30, with concentrations of 5% as used previously (Grégoire et al., 2012, Cairns et al., 2003) as our control group. Neurobion is usually administered by either oral administration or intramuscular injection (higher bioavailability) for 7–10 days continuously to treat acute or chronic pain clinically (Higashi et al., 2007). We chose a 10-day intraperitoneal (IP) Neurobion administration using a higher dose based on the clinical literature (Dimpfel et al., 1990, Calderon-Ospina et al., 2020). The Neurobion used in this study was obtained from a commonly available source and was prescribed by physicians in the clinic to simulate the closest condition of the clinic, as it was important to use a product that is representative of typical clinical practice and widely available to patients. The second group was therefore predosed with NEUROBION injected at 1 mL/kg of body weight [1 mL containing 33.3 mg B1, 33.3 mg B6, and 0.333 mg B12]. The injections were continued for 10 days, and were then injected with formalin (TG) on the last day (an hour after the last Neurobion injection) using the same protocol as group 1. To evaluate the effect of pain or stress following the intraperitoneal (IP) injection alone, we added a third (Vehicle) group, which like group 2 was acclimatized and handled for 10 days, but received saline intraperitoneally every day and one hour before the formalin test for the last time.

### 2.2 Pain measurement evaluation (pain score)

Formalin causes inflammation by injection into the plantar area of the rat's hind paw allowing us to evaluate how the animal responds to pain in both acute and chronic phases. Researchers suggest that formalin testing, compared to thermal and mechanical pain models, is one of the most reliable models for assessing clinical pain (Chiu et al., 2011), and it allows us to determine the acute and chronic phases in which Neurobion exerts its effect. It has been previously reported that a biphasic nociceptive behavior could be clearly observed after formalin injection (Salinas-Abarca et al., 2017); the first phase is known as the acute phase, which includes the first five minutes after injection. This phase is mainly caused by pain afferent fibers, mostly the C fibers through direct chemical stimulation. However, it is also accompanied by direct and strong stimulation of myelinated and non-myelinated fibers that represent neurogenic pain. The second phase starts from around minutes 10–35. This phase is a clear sign of an inflammatory response to tissue damage during the initial phase resulting in inflammation-driven pain. It can be characterized by the activity of non-myelinated fibers and hyperalgesia, which is caused by functional changes in the dorsal horn of the spinal cord as a result of central sensitivity (Salinas-Abarca et al., 2017). Interphase (the time between 5 and 10 min) separates these two phases and is thought to result from an active inhibitory process. Pain reduces after the second phase and during the recovery phase, from around minute 35–60.

Formalin was injected, and pain-related behavior (pain score) was accurately monitored for an hour and recorded every 15 s, continuously. The score was given to the rat’s behavior according to the following quantification of pain-related behavior (Abbott et al., 1995): Score zero was used when no pain-related behavior was observed, and the rat did not pay attention to the injected foot; Score 1 was used when the rat tried to walk or rest while putting most of his body weight on the non-injected leg; Score 2 was used when the rat lifted the injected leg; Score 3 was used when the rat was shaking or licking the injected foot. For pain intensity, the flinching and licking time and number were carefully observed for an hour and recorded every five minutes.

### 2.3 Spinal cord and brain tissue collection

For histochemical evaluation, four rats were randomly chosen from each group following behavioral observation. The spinal cord and brain tissue were fixed exactly two hours after the formalin injection (an hour after the end of the one-hour behavioral testing process). First, rats were deeply anesthetized by intravascular injection of Urethane (1.5 gr/kg). Subsequently, to perfuse and fix the brain and spinal cord tissue, the chest was opened and the pericardium and thymus were separated from the heart. Sodium nitrite (0.5 mL, 1%) and heparin (5000 IU) were injected into the left intraventricular space to open the vessels and prevent clot formation, respectively. Following the insertion of a cannula in ascending aorta, saline solution (500 mL) was circulated and the right atrium was cut with scissors subsequent to complete exsanguination. Fixative (containing 250 mL phosphate-buffered saline (PBS) (0.2 M, pH=7.4), 50 mL picric acid (1.33%) and 100 mL paraformaldehyde (20%) in 100 mL deionized water; 500 mL, 4 °C) was circulated for 3 h. The brain and spinal cord were carefully removed and stored in the same fixative for 24 h at 4 °C. Subsequently, the brain and spinal cord were immersed in 20% sucrose and PBS at 4 °C, followed by washing three times with PBS and freezing using liquid nitrogen. A Cryostat freezing microtome device (Leica, Germany) was used to cut the frozen tissues to thin sections of 40 µm, and the lumbar spinal cord and brain sections were kept in PBS at 4 °C.

### 2.4 Histochemical and Immunohistochemical analysis

For NADPH-diaphorase (NADPH-d) histochemistry, the lumbar spinal cord and brain stem sections were kept at room temperature and rinsed three times with PBS containing Triton X-100 (0.1%), then incubated in the PBS (0.1 M) containing Triton X-100 (0.3%), β-NADPH (0.1 mg/mL) and nitro blue tetrazolium (NBT) (0.1 mg/mL) with light protection at 37 °C for 1 h, till a purple color was observed. Once the reaction had completed, the sections were taken out and washed three times with PBS and deionized water, and mounted on glass slides, air-dried and cover-slipped with Entellan rapid mounting (Merck, Germany).

Alternate sections from the lumbar spinal cord and brain stem were used for immunohistochemical evaluation of *c-Fos*. The sections were rinsed three times (5 min) in PBST (Phosphate-Buffered Saline with Tween 20) followed by immersing into hydrogen peroxide solution (0.3%) for 30 min at room temperature and were washed two times again with PBST (5 min). After incubation in PBS solution containing 9% Bovine Serum Albumin (BSA) for 60 min at room temperature and washed again with PBST (5 min), the samples were incubated in PBST, BSA, Azide (3 mg), and primary antibody *Anti-c-Fos rabbit Ab* (Calbiochem, USA; RRID:AB_213451) diluted 1:10000 at 4 °C for 72 h. Sections were washed three times in PBST and incubated in a solution of BSA (3%), 3 mg of Azide, and biotinylated secondary antibody (Anti Rabbit IgG (whole molecule) (Sigma, USA; RRID:AB_916366), with the concentration of 1:1000 at 4 °C for 24 hr. After 3 washes with PBST the sections were placed in biotin peroxidase complex (ABC-vector) in PBST solution for 90 min. Following 3 further washes in PBS and tris-HCl buffer (0.05 M, pH7.4), the sections were placed for 10 min in Tris-HCl buffer containing Diaminobenzamide tetrahydrochloride (5 mg/mL), ammonium nickel sulfate (50 mg/mL), and hydrogen peroxide (0.5 µl/mL). The reaction was stopped by washing the sections in Tris-HCl buffer. Sections were then mounted onto glass slides, air-dried and cover-slipped. The *c-Fos* positive neurons turn dark blue with an ovaloid shape. An Olympus microscope was used to capture photos, and a camera lucida microscope was used for counting the neurons from both sides; at least 20 sections were taken from the lumbar region (L4 and L5) of each rat and at least five sections from the supra-spinal areas from each rat. The counting was performed using a microscope equipped with a grid system, and the cells were counted in a predetermined region of interest. We used the hypothalamic region as a control for *c-Fos* immunoreactivity after formalin paw injection.

### 2.5 Statistical analysis

All data are expressed as the mean ± SEM. Visualizations of our sample group data were plotted using Raincloud plots (Allen et al., 2021) and analysis was performed using JASP V0.16.3 (Team, 2022). We used the Shapiro-Wilk test to check the normality of our data distribution, and Levene’s test to check the homogeneity of variance between groups. For the behavioral results, where Levene’s test of homogeneity passed, we use a two-way ANOVA with tukey *post-hoc* correction. For groups that exhibited significant inhomogeneity (p < 0.001) we reverted to individual Welch’s tests (for non-equal variances) and Mann-Whitney U tests (for non-normal distributions). p values less than 0.05 indicated statistical significance. Where available we also calculated Cohen’s d to estimate the effect size, taking d > 0.2 = small effect, d > 0.6 = medium effect and d > 0.8 = large effect.

## 3 Results

The results of this research are separated into two parts; behavioral observation and histological investigation. For the behavioral observation, the formalin test was used for pain induction, and quantitative measurement of the number and duration of licking and flinching (along with a resultant pain score) compared between the CG and the pretreatment group. For histological investigation, exemplar rats were chosen randomly after behavioral observations. NADPH-d and *c-Fos* reactivity were used to investigate changes in the pain pathways, from the lumbar spinal cord to supra-spinal structures which are involved in pain processing.

### 3.1 Behavioral observations and quantification

**Fig. 1:**
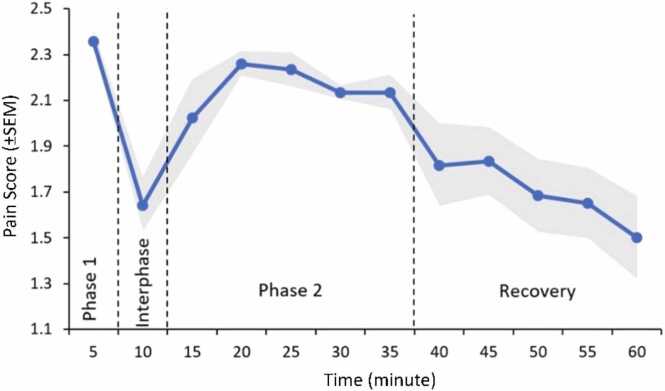
The separate phases of the pain response in formalin test. First phase, interphase, second phase and recovery time in the pain score of the control group (n = 6). The gray band in the figure represents ± SEM for the pain scores.

The biphasic nociceptive behavior could be clearly observed after formalin injection in our study. The first phase (phase I) is known as the acute phase, which includes the first five minutes after injection. The second phase (phase II) starts shortly after the phase I and lasts longer, around minutes 10–35. Interphase (the time between 5 and 10 min) separates these two phases. Pain reduces during the recovery phase, which lasts from around minute 35–60 (Fig. 1).

**Fig. 2:**
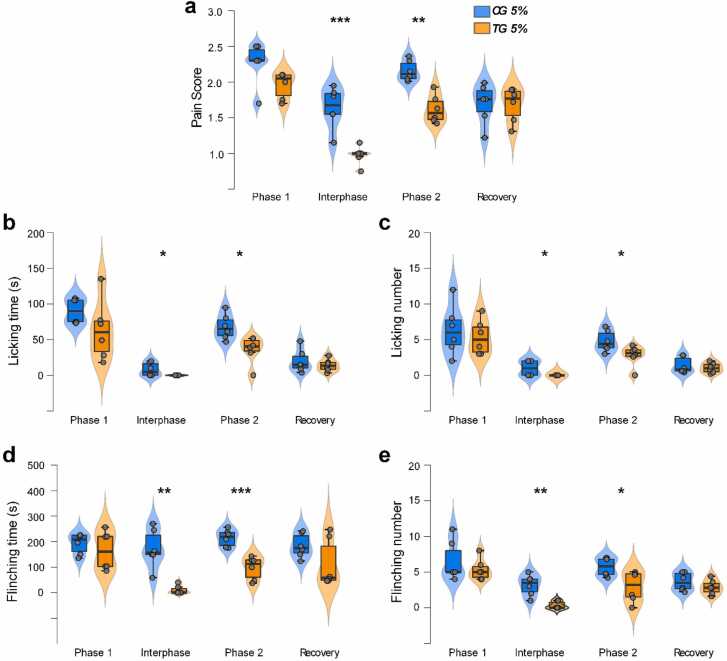
Distribution plot comparison of pain behavior evaluation between Neurobion pretreatment (TG) and its control (CG) in the formalin test. Significant decreases were most clearly seen in the composite pain score (a) and behavioral responses (b-d) for interphase and Phase 2 periods (n = 6, *P < 0.05, **P < 0.01, and ***P < 0.001).

Behavioral evaluations were performed for the control group (CG) and the Neurobion pretreatment group (TG) across the four time periods illustrated in Fig. 1. A two-way ANOVA showed that there were significant and large difference between CG and TG (F(1, 32) = 33.22, p < 0.001; effect size ω₂ = 0.19), a significant and large difference across time period (F(3, 32)= 26.58, p < 0.001; effect size ω₂ = 0.46), and a significant and medium-sized interaction (F(3, 32) = 4.72, p = 0.006; effect size ω₂ = 0.07). Simple main effects analysis showed that there were significant differences between CG and TG for phase 1 (p = 0.025), interphase (p < 0.001) and phase 2 (p < 0.001); but no significant difference for recovery (p = 0.930). *Post-hoc* corrected multiple-group comparisons confirmed highly significant differences between CG and TG for interphase and phase 2 only (Fig. 2a; p < 0.001 & p = 0.005 respectively).

Decreases in the respective behavioral response composites were seen for all phases of both licking time and licking number; these reached significance in the interphase (*licking time*: p = 0.046 Student’s T; Cohen’s d = 0.85) and the second phase (*licking time*: p = 0.014 Student’s T, Cohen’s d = 1.72 ± 0.76; *licking number*: p = 0.04 Student’s T, Cohen’s d = 1.36 ± 0.7) for TG compared to CG (Fig. 2 b and c). Fig. 2 d and E plots flinching time and flinching number, which clearly show significant reductions in TG in both the interphase (*flinching time*: p = 0.003 Welch’s T, Cohen’s d = 2.94 ± 1.03; *flinching number*: p = 0.004 Welch’s T, Cohen’s d = 2.57 ± 0.94) and the second phase (*flinching time*: p = 4.3 ×10⁻⁴ Student’s T, Cohen’s d = 2.97 ± 1.03; *flinching number*: p = 0.026 Welch’s T, Cohen’s d = 1.57 ± 0.73).

To summarize the behavioral assessment, Neurobion resulted in significant reductions of pain in the second phase and interphase but not in the first phase and recovery time, which indicates that the greatest effect of Neurobion is in the chronic phase of the pain response.

**Fig. 3:**
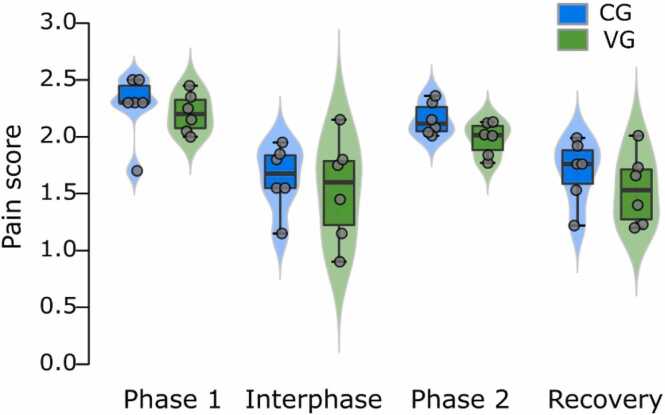
Comparison of pain score between Vehicle group (VG) and control group (CG) in the formalin test. The mean pain score has not any significant difference (n = 6).

In order to monitor the effect of the needle pain caused by the IP injection of Neurobion, a Vehicle group (VG) received the saline followed by a 5% formalin injection (after 1 h). The result obtained from this group was the same as the CG (Fig. 3).

### 3.2 Histochemical analysis

For histochemical and immunohistochemical evaluation, we utilized at least 20 sections from the spinal cord (the focus was on L4 and L5 of the lumbar region) and five sections were obtained from each rat's supra-spinal areas of interest.

**Fig. 4:**
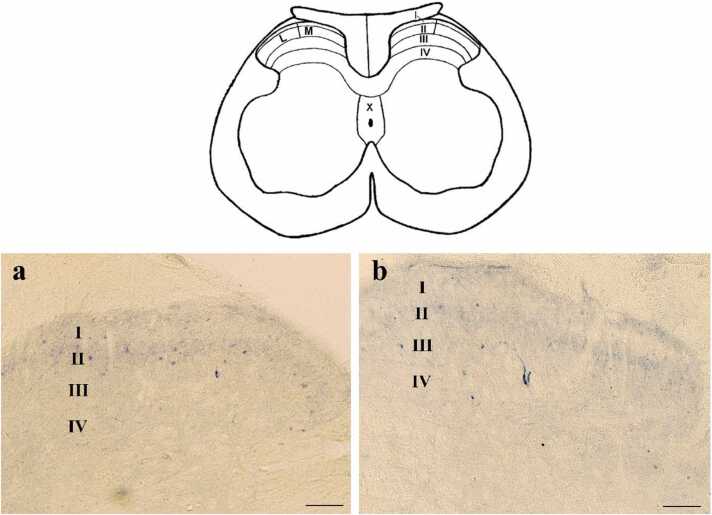
Schematic diagram (upper) and Photomicrograph of NADPH-d positive neurons (lower) in lumbar spinal cord**.** The Schematic diagram shows the white and gray matter of the spinal cord and its dorsal and central horn laminae. Roman numerals in the schematic figure indicate the corresponding laminae. M, shows the medial side and L shows the lateral side of the dorsal horn. The Photomicrograph of NADPH-d positive neuron shows the dorsal horn laminae of the CG (a) and TG (b) groups. (Scale bar = 100 µm).

To assess the NADPH-d positive neurons in this study, we investigated the dorsal horn region that contains laminae I to IV, and also areas around the central canal, including lamina X. Laminae I - II as superficial laminae of the dorsal horn and laminae III - IV as deep laminae were examined separately. The mentioned above classification is shown schematically in the schematic diagram (Fig. 4).

#### 3.2.1 Number of NADPH-d positive neurons in the dorsal horn

**Fig. 5:**
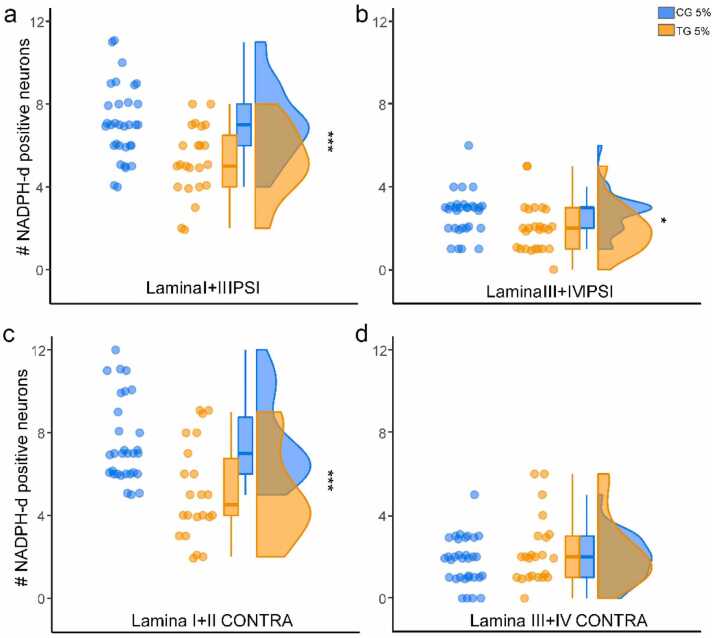
The NADPH-d positive neurons after formalin injection in superficial and deep laminae of the dorsal horn in CG and TG. The population plots show the number of NADPH-D positive neurons in the laminae of the dorsal horn ipsilateral to (a and b) and contralateral to (c and d) the injected side. (*P < 0.05 and ***P < 0.001).

In the superficial laminae of the dorsal horn, the small axons had a spindle-shaped cell body and appendages that projected a short distance around neurons. In the deep laminae of the dorsal horn, larger neurons with a multipolar cell body were seen, with their appendages branching out in all directions. Extensions of some of these neurons also extended to the superficial laminae. Following the formalin injections, the number of NADPH-d positive neurons in the superficial and deep laminae of the dorsal horn was investigated in CG and compared with TG in the injected side and the opposite side of the injection. After the formalin injection, the number of NADPH-d positive neurons of the TG group was significantly lower than CG in the dorsal horn laminae of the spinal cord, especially in the superficial laminae (I – II) on both sides (Fig. 4a and b and Fig. 5).

In the superficial laminae (I and II), we observed a 25% decrease (SD=0.4) in the number of NADPH-d positive neurons on the injection side (Laminae I+II IPSI, p < 0.001, Student's T-test; effect size 1.05 ± 0.3). The mean number of neurons on the injection side was 7.1 ± 0.3 (mean ± SEM) for the CG and 5.2 ± 0.3 for the TG. On the opposite side of the injection (CONTRA I+II), we observed a 15% decrease (SD=0.6) in the number of neurons (p < 0.001, Mann-Whitney U test; effect size 0.30 ± 0.7). The mean number of neurons on the opposite side was 7.5 ± 0.3 for CG and 5.1 ± 0.4 for TG.

Regarding the deep laminae (III and IV), we observed a 34% decrease (SD=0.4) in the number of neurons on the injection side (Laminae III+IV IPSI, p = 0.013, Mann-Whitney U test; effect size 0.37 ± 0.1). The mean number of neurons on the injection side was 2.7 ± 0.1 for CG and 2.0 ± 0.2 for TG. However, there was no significant difference in the number of neurons on the contralateral side (CONTRA III+IV, p = 0.38, Mann-Whitney U test). The mean number of neurons on the contralateral side was 1.8 ± 0.2 for CG and 2.2 ± 0.3 for TG. (Fig. 5 **a-d**).

#### 3.2.2 The number of NADPH-d positive neurons in lamina X

**Fig. 6:**
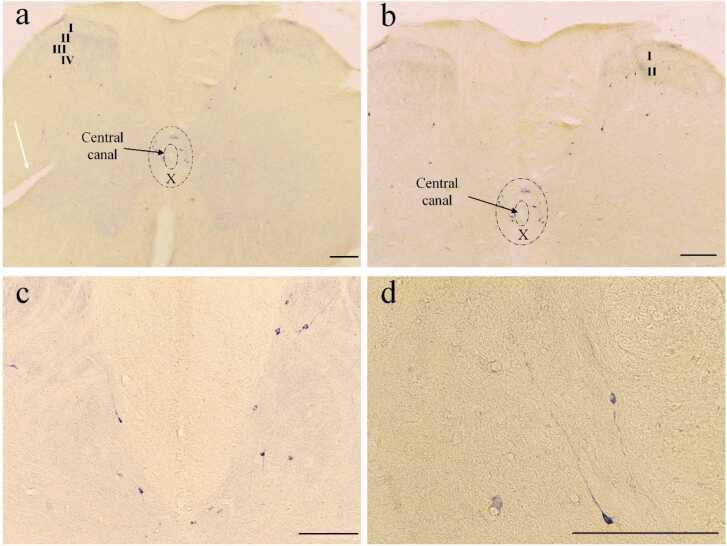
NADPH-d positive neurons in the dorsal horn and X lamina. (a) and (b) represent spinal cord sections and activated neurons in the dorsal horn of the injection site in the control group. (c) NADPH-d positive neurons whose axons extend from the central canal (lamina X) to the dorsal horn. (d) neurons at a higher magnification. The white arrow in (a) indicates the knife cut determining the side opposite the injection. (Scale bar = 200 µm).

The NADPH-d positive neurons in lamina X contain bigger cell bodies and indicate a variety of morphologies. It was observed that some of the neuronal appendages extended into the dorsal horn (Fig. 6).

**Fig. 7:**
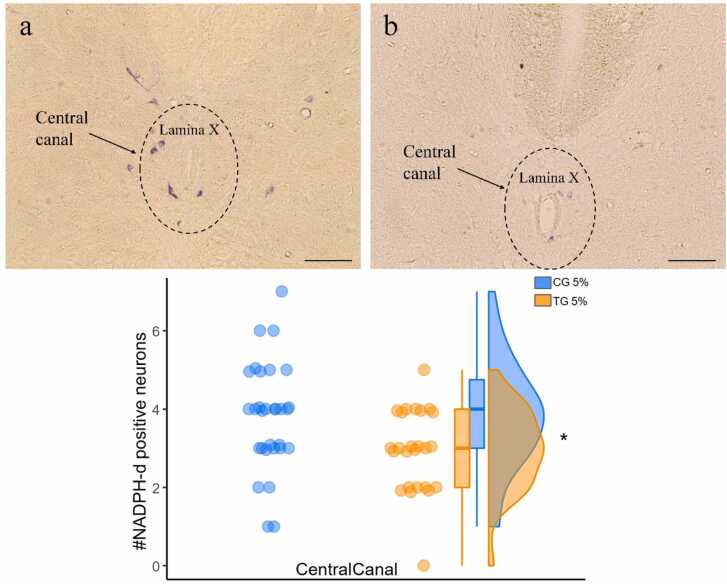
Photomicrograph of lumbar spinal cord sections of positive NADPH-d neurons around the central canal, in CG (a) and TG (b). The raincloud plot (c) shows the positive NADPH-d neurons on the injected side of the central canal in CG and TG groups after formalin injection. (Scale bar=100 µm) (*p < 0.05).

Around the central canal (lamina X), the number of positive NADPH-d neurons in the upper-quarter of the injected area (synapsing on the same side as the dorsal horn) demonstrated a significant (p = 0.016 Mann Whitney) reduction in the number of positive NADPH-d neurons in TG compared to CG. For the CG, the mean number of positive NADPH-d neurons was 3.8 ± 0.2 (mean ± SEM). In contrast, the TG showed a lower mean number of positive NADPH-d neurons, with a value of 2.9 ± 0.2 (mean ± SEM). (Fig. 7).

#### 3.2.3 The number of NADPH-d positive neurons in supra-spinal areas

**Fig. 8:**
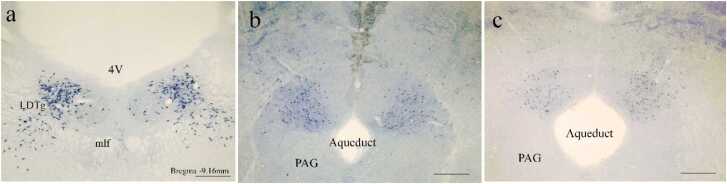
NADPH-d positive neurons in the supra-spinal areas. (a) The positive NADPH-d neurons (in purple) in LDTg (a) and dorsal area of the PAG in CG (b) and TG (c). There was no significant change on either side of the injection and in both control and Neurobion pretreatment groups. (Scale bar=500 µm). LDTg: laterodorsal tegmental nucleus, mlf: medial longitudinal fasciculus, 4 V: 4th ventricle, and PAG: Periaqueductal gray.

The supra-spinal areas focused on for histochemical evaluation in this study were the PAG, parabrachial nucleus, and laterodorsal tegmental nucleus (LDTg). However, given the high density of neurons in these areas in multiple layers, it was impractical to count them accurately with a camera lucida, and visual observation showed no change on the injected side compared with the noninjected side and also for TG compared with CG (Fig. 8 a-c).

### 3.3 Immunohistochemistry reaction of c-Fos protein

**Fig. 9:**
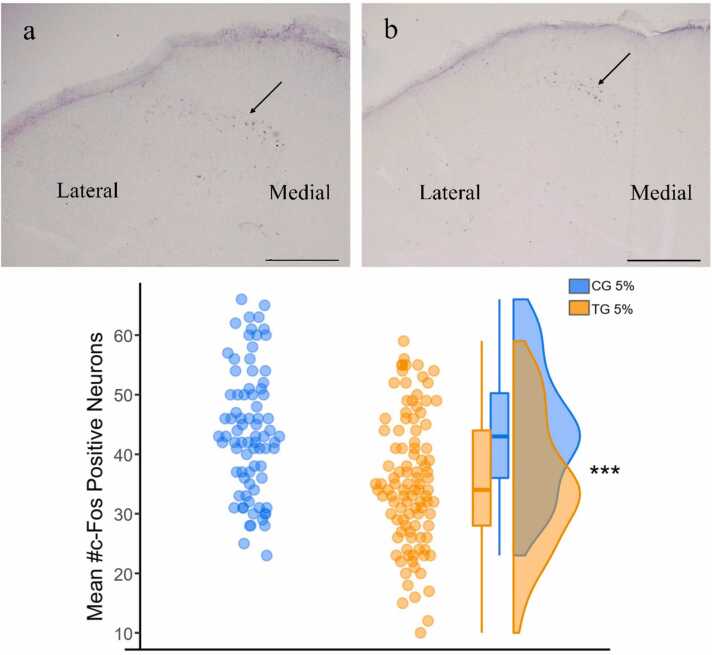
Immunohistochemical reaction of c-Foc protein in the superficial laminae (I – II) in CG and TG groups. Microscopic images indicate the dorsal spinal cord in the injected side of CG (a) and TG (b) and the mean difference in their neuron number is shown in chart (c). The black arrows indicate *c-Fos* positive neurons. (***P < 0.001).

The immunohistochemical reaction of c-Fos protein in the lumbar area of the dorsal spinal cord and supra-spinal centers was also investigated in response to the inflammatory pain induced by formalin. The results indicated that positive *c-Fos* neurons in the dorsal spinal cord of both CG and TG were observed just in the injected side (Fig. 9a, b). In the superficial laminae (I - II), there was a highly significant reduction in the number of positive *c-Fos* neurons for the TG compared to the CG (p = 5.03 ×10⁻⁷, Student's T-test; effect size 0.78 ± 0.16).

Specifically, the mean number of positive *c-Fos* neurons in laminae (I - II) for the TG was 35.3 ± 1.0, while the mean number for the CG was 43.8 ± 1.0 (mean ± SEM).

## 4 Discussion

In this study, the effect of chronic Neurobion pretreatment on an inflammatory pain test was investigated using both behavioral and morphological methods. To our knowledge, this is the first study comparing B vitamin treatments that utilize NADPH-d and *c-Fos* to evaluate the potential role of NO in the pain pathway in the dorsal horn of the spinal cord and supra-spinal centers.

We found that Neurobion pretreatment resulted in a clear and significant reduction of behavioral responses to pain in the chronic phase. This result is in accordance with studies, which reported that chronic pretreatment of B-complex could decrease the pain response behavior i.e., the licking time (Reyes-García et al., 2002) and flinching scores (Reyes-García et al., 2006) in response to pain induced by the formalin injection model.

B-complex analgesic effects could potentially be mediated by various mechanisms. One possible mechanism is the modulation of neurotransmitter systems involved in pain processing. B vitamins, such as vitamins B6 and B12, play essential roles in the synthesis and metabolism of neurotransmitters, including serotonin, norepinephrine, and gamma-aminobutyric acid (GABA). These neurotransmitters are involved in pain modulation, and alterations in their levels or activity can influence pain perception. Therefore, supplementation of B-complex may restore or enhance the functioning of these neurotransmitter systems, resulting in reduced pain responses.

Furthermore, B-complex vitamins are reported to possess anti-inflammatory properties. Inflammation plays a crucial role in the development and maintenance of pain, and reducing the inflammatory response can alleviate pain symptoms. B vitamins, such as vitamin B3 (niacinamide), have been shown to exhibit anti-inflammatory effects by inhibiting the production of pro-inflammatory cytokines and modulating immune cell activity (Surjana et al., 2010). Therefore, it is plausible that the anti-inflammatory actions of B-complex contribute to its analgesic effects in the formalin-induced pain model.

In addition to behavioral responses, it is important to consider the neural correlates of the observed analgesic effects. Previous studies have shown that formalin-induced pain is associated with specific neuronal activation, which is believed to play a role in pain transmission and modulation.

Our results indicated that formalin injection in rats’ right paws could increase the number of the NADPH-d positive neurons on both sides of the dorsal horn at the lumbar level of the spinal cord as well as in the X lamina. This observation aligns with previous studies highlighting the involvement of NADPH-d positive neurons in pain processing (Rodella et al., 1998, González et al., 2001). Furthermore, regulatory function of Vitamin B in the synthesis of neuroactive substances is well-documented that Vitamin B plays a crucial role in the regulatory of various neuroactive substanc-es, including nitric oxide (NO) (Weinberg et al., 2009). More interestingly, after formalin injection in the Neurobion pretreated group, the number of NADPH-d positive neurons on both sides of the superficial laminae in the dorsal horns showed a significant decrease compared to the control group. The same pattern was observed in the area around the central canal. This finding suggests that Neurobion, which contains essential B vitamins, may modulate the syn-thesis or release of neuroactive substances involved in pain processing, such as NO.

Studies have shown that neurons containing NOS in these laminae are actually spinothalamic projection neurons involved in spinal cord pain processing, and bilaterally activation of these neurons in the spinal cord has been reported following inflammation caused by formalin (Tsuruoka et al., 2003) or carrageen (Traub et al., 1994) injection in a rat’s paw.

Considering the reduction of pain behavior alongside the reduction of the number of NADPH-d positive neurons in the superficial laminae of the dorsal horn and lamina X, it is reasonable to assume reduced pain is associated with changes in NO mediated neural transmission. Supporting this view following formalin injection in the hind paw of the rat, the number of neurons that express nNOS (Neuronal nitric oxide synthase) in the lumbar of the spinal cord increases (Traub et al., 1994), along with blocking NOS by L-NG-Nitroarginine Methyl Ester (L-NAME) reduced the effect of B vitamins, which suggests that B vitamins exert some of their analgesic effects by releasing NO (Reyes-García et al., 2006).

It has been also shown that stimulation of primary sensory neurons by a variety of painful stimulations causes rapid observation of the expression of the c-Fos protein in the nucleus of post-synaptic neurons in the superficial layers of the dorsal horn of the spinal cord unilaterally, which is used as an indirect marker of neuronal activity in determining pain pathways.

Some studies have investigated the relationship between Fos-positive/NADPH-d neurons and pain behaviors in formalin-induced pain conditions. The researchers observed a positive correlation between the number of Fos-positive/NADPH-d neurons and pain response behaviors, such as licking and flinching, suggesting a potential involvement of these neurons in pain transmission or modulation (Polgár et al., 2013, Cao et al., 2000).

It has been reported that the distribution and activation patterns of Fos-positive/NADPH-d neurons differed between the acute and chronic phases of pain (Hossaini et al., 2014). In the acute phase, Fos-positive/NADPH-d neurons were predominantly observed in areas associated with pain transmission, suggesting their involvement in the immediate response to pain. In contrast, in the chronic phase, Fos-positive/NADPH-d neurons were more widely distributed, including areas associated with pain modulation, indicating their potential role in the long-term processing and modulation of pain. These findings highlight the importance of investigating the relationship between Fos-positive/NADPH-d neurons, pain behaviors, and pain pathways in the context of formalin-induced pain. The activation and distribution patterns of these neurons provide valuable insights into the neural mechanisms underlying pain perception and modulation.

The expression of *c-Fos* pattern in visceral stimulations is also different from its pattern in somatic stimulation which expresses bilaterally (Honoré et al., 1995). Expression of *c-Fos* after stimulation of pain receptor neurons in the spinal cord can be stopped by analgesics and pain killers such as morphine, Indomethacin (Bullitt, 1990) and ketoprofen (Roche et al., 1996) before carrageenan test. Each of these demonstrations confirms the relation between *c-Fos* expression and pain. In our study, we also observed a significant decrease in the number of *c-Fos* positive neurons in the posterior spinal cord laminae in the Neurobion pretreatment group compared to the control group. However, studies have shown that *c-Fos* is still expressed after anesthesia which reduces pain or pain responses. It has been reported that after pain relief, *c-Fos* expression is still high, which indicates that *c-Fos* expressing cells are still sensitive, and there are theories that *c-Fos* expression represents central sensitivity.

It has been previously suggested that NO-producing cells may have an intrinsic property that creates a lower threshold for the expression of *c-Fos* than other neurons. It is also possible that NO released from the cellular body or axon-dendrites of these neurons induces the *c-Fos* expression by affecting nearby neurons (Fukuda et al., 2006). However, Todd et al. have reported that after noxious stimulation, c-Fos expression is observed in various types of spinal neurons, including projection cells, excitatory interneurons, and both excitatory and inhibitory interneurons (Todd et al., 1994). It seems that licking, as a non-painful stimulation, affects the threshold of nearby neurons by stimulating primary sensory neurons, therefore, increasing the expression of *c-Fos* in painful situations. The *c-Fos* positive cells may contain some interneurons containing inhibitory neurotransmitters such as GABA, glycine, enkephalin, or dynorphin. It is probably possible that licking plays an important role in modulating the expression of *c-Fos* in the dorsal horn of the spinal cord, especially in the superficial laminae on the same side.

There is also evidence suggesting the role of *c-Fos* neurons in modulating hyperalgesia. Hunter et al. reported the importance of *c-Fos* in the formalin test, which is necessary to reduce pain (possibly by the mechanism of dynorphin) (Hunter et al., 1995). During hyperalgesia, the adjustment or compensation mechanism is up-regulated. Therefore, the nervous system tries to weaken hyperalgesia with the Fos mechanism. This explains why L-NAME reduces *c-Fos* because it reduces progression by the NMDA-NOS knockout mechanism and suggests that NO may be involved in understanding chronic pain by strengthening the activity of spinal cord pain receptor neurons by the NMDA receptor-dependent mechanism. It is also suggested that NO be involved in the production of Fos protein. Therefore, the use of NO-blocking agents may be a way to inhibit the progression of hyperalgesia after inflammation and perhaps even after peripheral nerve injury.

Combining previous works with our study's findings, the observed reduction in c-Fos expres-sion in the dorsal spinal cord after Neurobion pretreatment, alongside its increased expression aligning with pain indices, suggests a potential involvement of c-Fos in pain induction, consistent with existing literature. Furthermore, our results suggest that Neurobion may exert analgesic ef-fects through mechanisms akin to painkillers that utilize NO-blocking agents in hyperalgesia or painful situations. By reducing the number of active neurons, that utilize NO as a pain neuro-transmitter, Neurobion could potentially contribute to pain reduction. These findings indicate the potential of Neurobion as an adjuvant with painkillers to enhance pain relief or as a standalone treatment for alleviating pain.

Some studies also have suggested that the analgesic effect of some painkillers is due to the activation of descending inhibitory pathways. As, a recent study (Hashemi et al., 2022) has shown that high levels of NO in the dorsolateral periaqueductal gray (dlPAG) may regulate the pain process through downward synaptic interactions. The involvement of NO in both the dorsal hippocampus (dH) and dlPAG matter was observed in morphine-induced analgesia during the rat formalin test. Injection of L-arginine, reduced the morphine response in both stages, while the administration of L-NAME, the nNOS inhibitor, had no effect. However, when L-NAME was injected into the dlPAG prior to L-arginine at the dH, the morphine-induced response was reinstated in the early stages, indicating the modulation of NO in the pain pathway from the dH to the dlPAG. In another study that used the formalin test and then evaluated the effect of intracerebroventricular injection of enkephalin, the results demonstrated that pain score decreased, and *c-Fos* expression in laminae related to pain processing in the spinal cord was inhibited (Hossaini et al., 2014). This experiment is strong evidence for the theory that supra-spinal opioids reduce pain by activating descending inhibitory control. In particular, the activation of PAG neurons stimulates neurons in the raphe nuclei, which synapse with the sensory neurons in the spinal cord and inhibit them. This study suggests that the descending inhibitory mechanism (which inhibits the firing of the neurons responding to pain), that acts at the spinal cord level is essential for pain-reducing behavior in the formalin test. Bilateral injury on the posterior surface of the pectoral spinal cord prevented pain relief and reduced the *c-Fos* expression of intracerebroventricular injection of enkephalin.

Although we were not able to show the effect of pain and its reduction by Neurobion in the supra-spinal region using histochemical and immunohistochemical reactions, as licking is a supra-spinal behavior, it can be concluded that Neurobion can play a part of its analgesic role by affecting these areas. On the other hand, there are positive NADPH-d, not only in areas of the brain that are associated with the pain pathway, such as the hypothalamus, gray matter around the central aqueduct, reticular nucleus, Raphe Magnus, and Locus coeruleus (Lee et al., 1993), but also in other areas that are not involved in pain, such as tegmental nuclei, superior and inferior colliculus, pedunculopontine, hippocampal gyrus, supraoptic nucleus and striatum. This indicates a clear role of NO in the transmission and processing of other information in addition to pain across differing brain areas. Therefore, due to the different roles of supra-spinal regions, it prevents observation and measurement of the pain reduction effect of Neurobion without the investigation of other factors (Bredt et al., 1991).

## 5 Conclusion

Our results suggest that B-complex pretreatment can reduce the chronic phase of pain. Downregulation of *c-Fos* and NADPH-d in dorsal spinal neurons in the vitamin-treated group suggested that the antinociceptive effect induced by Neurobion could be due to a suppression of nociceptive transition at the spinal level, especially at the afferent region in the lumbar section of dorsal spinal horn. At least a part of the analgesic effects of Neurobion could be the result of decrement in the number of active neurons after pain inducement, in the site of afferent fibers entering the spinal cord. Those active neurons use NO at least as one of their pain neurotransmitters and reductions in their activity correlate with a decrease in the behavioral pain measure.

## Funding

The author(s) disclosed receipt of the following financial support for the research, authorship, and/or publication of this article: This work was supported by the 10.13039/501100001809National Natural Science Foundation of China [grant number 32070992], and Shahid Beheshti Medical University [grant number 1390288].

## CRediT authorship contribution statement

**Shahab A. Zarei**, Conceptualization*,* Experimental section, Data collection and analysis, Investigation, Methodology, Writing – original draft, review and editing, and incorporated feedback from all authors; **Mina Shahriari-Khalaji**: Writing – original draft, Writing – review & editing, Visualization, Design of graphical abstract; **Ian Max Andolina**: Supervision, Formal analysis, Data analysis curation, wrote parts of the manuscript and provided feedback on manuscript drafts, Writing – review & editing, Visualization; **Gila Behzadi**: Supervision, Conceptualization, Material preparation, Curation, Investigation, Methodology, Data monitoring, Writing – original draft, Provided feedback on manuscript drafts; All authors read and approved the final manuscript.

## Declaration of Competing Interest

The authors declare that there is no conflict of interest.
